# New Germline TP53 Variant Detected After Radiotherapy-Induced Angiosarcoma of the Chest Wall in a Previously Treated Breast Cancer Patient: A Case Report and Review of Li–Fraumeni Syndrome and Radiotherapy-Induced Sarcoma

**DOI:** 10.1155/crom/6640468

**Published:** 2024-12-19

**Authors:** Bruna Bianca Lopes David, Sávio Solon Alves Silva, Vanessa Dinoa, Tadeu Diniz, Emilio Pereira, Carolina Bustamante, Bernardo Garicochea

**Affiliations:** ^1^Medical Oncology Department, Oncoclinicas Group, Rio de Janeiro, Brazil; ^2^Clinical Research Department, National Cancer Institute (INCA), Rio de Janeiro, Brazil; ^3^Radiology Department/Surgical Orthopedics Department, Rio de Janeiro Federal University (UFRJ), Rio de Janeiro, Brazil; ^4^Surgical Orthopedics Department, National Institute of Traumatology and Orthopedics (INTO), Rio de Janeiro, Brazil; ^5^Pathology Department, Oncoclinicas Group, São Paulo, Brazil

**Keywords:** angiosarcoma, breast cancer, Li–Fraumeni syndrome, radiotherapy, sarcoma

## Abstract

Li–Fraumeni syndrome (LFS) is one of the most common hereditary cancer predisposition syndromes in Brazil. The high frequency of the syndrome is due to a founding variant (R337H) in the country. LFS is characterized by a wide variety of malignant phenotypes. Despite the great epidemiological importance of the R337H variant, the frequency of other types of pathogenic variants is like other populations, with the majority of these being missense variants. There is strong evidence that radiotherapy is associated with secondary sarcomas, including angiosarcomas, and this finding is especially true for LFS patients. Angiosarcoma is not described as overrepresented in individuals with LFS, except in patients submitted to radiotherapy. Germline testing in all breast cancer patients under 65 will reveal many germline mutations in TP53 without a family history of cancers associated with the syndrome. We present a case of a previously undescribed pathogenic variant in TP53 (c788del, pAns263llefs⁣^∗^82) in a patient with no family history of cancer, with a previous diagnosis of breast carcinoma treated with radiotherapy, who developed angiosarcoma after a few years leading to germline testing. The presence of angiosarcoma in a radiotherapy bed should raise suspicion for LFS. The recent recommendation of testing breast cancer patients under the age of 65, even without any family history, can be a source of discoveries of new mutations and assist in therapeutic decisions.

## 1. Introduction

Li–Fraumeni syndrome (LFS) is an autosomal dominant condition that originates from germline mutations in the TP53 gene. The incidence of prevalence of carriers in the world population is estimated at 1/5000 to 1/20,000 [1]. Its clinical presentation is marked by an important predisposition to the development of malignant neoplasms at an early age. The described syndrome in 1969 by Li and Fraumeni was characterized by the presence of five types of cancer: sarcoma, adrenocortical carcinoma (ACC), breast cancer, leukemia, and brain tumor [2]. The tumor suppressor gene TP53, located on chromosome 17p13, encodes a nuclear protein (p53) that controls multiple pathways of cell proliferation, homeostasis, cell cycle, apoptosis, and DNA repair. Germline mutations generally occur in Exons 3–9, located in preferential regions (hot spots), altering the structure and function of the p53 protein [3].

## 2. Diagnosis of LFS

The process of identifying individuals and families at risk of carrying the syndrome initially considered three classical criteria, based on the analysis that first described the LFS (Table 1). However, when a broader tumor spectrum was recognized, there was a need to develop new sets of criteria to identify patients with the syndrome, as the Chompret criteria and others—for those who did not meet the Chompret criteria. Thus, terms such as LFS-like and broader inclusion criteria, known as Birch and Eeles criteria, emerged (Table 1) [4–6]. The new set of criteria continue to have value in clinical practice, but since it was demonstrated that 80% of these cases carried germline mutations in TP53, a much larger number of cases have been identified, including new mutations and less penetrant phenotypes [7].

## 3. LFS and Predisposition to Cancer

Carriers of pathogenic germline variants in TP53 have a variable lifetime risk of developing cancer. In childhood (0 to 15 years), the predominant cancers are adrenal carcinoma, medulloblastoma, and rhabdomyosarcoma. In young adults, 51% of cancer cases are predominantly breast cancer, osteosarcoma, glioblastoma, hematological tumors, colorectal cancer, lung cancer, and soft tissue sarcomas. In elderly, cases of pancreatic and prostate cancer predominate.

**Figure 1:**
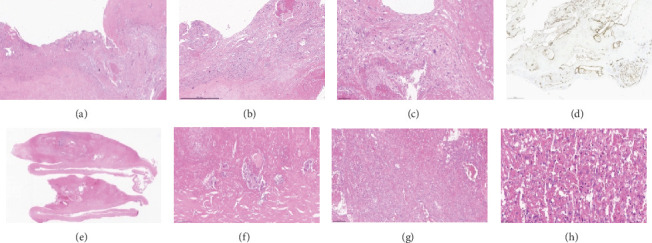
(a) Right breast prosthesis capsule with crystals and polyurethane. (b) Vascular channels and atypical neoplastic cells in the wall of the breast prosthesis capsule. (c) Atypical neoplastic cells and polyurethane crystals. (d) Immunohistochemistry for CD34 highlighting the vascular channels. (e) Connective tissue from the breast implant capsule with thickening area, H&E 2×. (f) Breast prosthesis capsule with fibrosis and giant cell foreign body reaction, involving polyurethane crystals. (g) Neoplastic cells forming anastomosing channels in the connective tissue. (h) Atypical neoplastic cells among red blood cells.

In Brazil, there is a higher than global incidence of the syndrome due to the presence of the founding mutation c.1010G>A; p.Arg337His (p.R337H) [8]. This variant entails a lifetime cancer risk of 80% in women and 47% in men. The p.R337H variant has a peculiar location in the gene, located in the C-terminal regulatory domain, a region with a very low concentration of pathogenic changes [8]. Most pathogenic variants are situated in the DNA-binding domain (DBD) between Exons 5 and 9 (Figure 1). The use of germline multigene panels has exponentially increased the diagnosis of LFS carriers and, as a result, the prognosis of these patients has improved considerably with screening programs that are constantly revisited.

## 4. The TP53 Gene

The TP53 gene, which encodes the p53 protein, is the most frequently altered gene in human neoplasms. TP53 acts as a transcriptional activating factor, regulating the expression of downstream genes through specific binding sites on DNA. Its best-known functions include antiproliferative effects such as inducing cell cycle arrest, DNA repair, cellular senescence, and cell death by apoptosis in case of irreparable DNA damage [9]. During damage events, activated p53 protein triggers repair mechanisms, resulting in the inhibition of cell proliferation and cell cycle arrest [9]. Consequently, it also prevents the accumulation of genetic variants by eliminating damaged cells, being referred to as “the guardian of the genome” [9].

The TP53 gene, represented in Figure 1, is composed of 11 exons, with the first being noncoding. The p53 protein, a tetrameric phosphoprotein, is composed of 393 amino acid monomers, organized into five distinct functional domains. These include a transactivation domain at the amino-terminal end (Residues 20–62), responsible for the activation of target genes; a proline-rich domain (Residues 63–97), essential for interactions with apoptosis-inducing proteins; a DBD in the central region (Residues 102–292), evolutionarily conserved and responsible for promoting the binding of p53 to DNA consensus sequences present in the promoter regions of its target genes; an oligomerization domain (OD) (Residues 323–356), necessary for dimer formation and subsequent formation of p53 homotetramers; and a carboxy-terminal domain, implicated in the regulation of DNA binding (Residues 363–393) [9].

In contrast to other tumor suppressor genes, which are predominantly altered by truncating variants, most germline alterations in TP53 are of the missense type, according to the IARC (International Agency for Research on Cancer) database [10]. These variants are mainly concentrated in the DBD, resulting in the loss of specific DNA binding. In addition to the DBD, less common variants are found in the OD and reduce not only the DNA binding affinity of p53 but also its ability to recruit other transcription cofactors. Both properties are essential for the p53 protein to exert precise and efficient transcriptional control over its numerous target genes.

## 5. Case Report

The patient is a 47-year-old female, with a previous history of bilateral breast cancer at the age of 34. At that time, bilateral mastectomy was indicated, with left axillary lymph node dissection. The anatomopathological report showed invasive ductal carcinoma on the right, negative hormone receptors, positive HER2 receptor (Score 3+), pT1pN0, and, on the left side, no special ductal carcinoma, negative hormone receptors, positive HER 2 (Score3+), pT2pN1, with positive sentinel lymph node testing. The left breast also had associated Paget's disease. After surgical resection, adjuvant chemotherapy was given followed with docetaxel and cyclophosphamide, associated with trastuzumab for six cycles and maintenance with trastuzumab until completing 14 cycles. In the meantime, the patient received radiotherapy to the bilateral breast plastron with 25 fractions and a total of 50 Gy. She remained under clinical surveillance and had breast reconstruction with prostheses later.

After 13 years, symptoms of progressive discomfort with the bilateral breast implants surfaced, associated with the perception of thickening in the lower quadrants. Bilateral breast ultrasound showed suspicious images of breast nodules measuring 2.7 cm × 1.0 cm in the lower quadrant of the left breast and 1.8 cm × 0.9 cm in the lower quadrant of the right breast. Complementary magnetic resonance imaging of the breasts showed only one nodule in the left breast, measuring 2.9 × 2.6 × 1.1 cm, which adhered anteriorly to the breast prosthesis capsule.

A bilateral biopsy was performed which revealed spindle cell neoplasia in both injuries close to the prostheses. Slide reviews were requested and confirmed the diagnosis of bilateral mammary angiosarcoma, with the following immunohistochemical findings: FLI-1 positive, CD31 positive, CD34 positive, and ki67 positive in 40% of the sample (Figure 1). MYC amplification research was not performed. Genetic counseling and subsequent germline blood testing for a panel of genes associated with hereditary cancer syndromes showed a pathogenic variant for LFS (TP53 gene mutation—c.788del; p.Asn263Ilefs⁣^∗^82/het/rs1597362423), a variant not yet described. Staging with PET-CT did not reveal metastatic disease, only nonspecific pulmonary nodules. Laboratory test showed no significant changes. Among the relatives investigated, including parents, siblings, and first-degree cousins, none presented a pathogenic p53 variant in genetic screening, indicating a de novo case.

After a multidisciplinary discussion, neoadjuvant chemotherapy with doxorubicin 75 mg/m^2^ and ifosfamide 9 g/m^2^ was deliberated for six cycles, given the aggressiveness and bilateral nature of the angiosarcoma.

**Figure 2:**
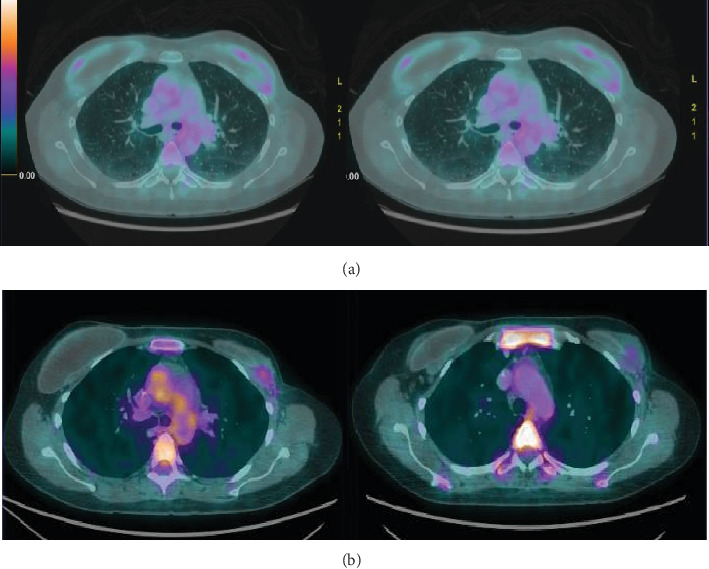
PET-CT scan during systemic treatment shows reduction of nodules and absence of contrast uptake. (a) Before starting chemotherapy. (b) After six cycles of chemotherapy.

During the systemic treatment, clinical and radiological response was observed (Figure 2). There were no serious complications during chemotherapy. After six cycles, the patient underwent surgical treatment with removal of the breast prostheses and breast capsules and dissection of musculocutaneous planes included in previous radiotherapy. The histopathological report was compatible with a complete pathological response bilaterally. A new course of radiotherapy for local control was not indicated.

The patient has been undergoing clinical and radiological control for 2 years with whole-body MRI and chest CT scan at a low radiation dose. Currently, she is asymptomatic, and there is no evidence of disease.

## 6. Discussion

LFS is an autosomal dominant condition that predisposes to a high risk of developing cancer. Germline pathogenic variants in the TP53 gene are the underlying cause of the syndrome. Identifying families with p53 mutations is a difficult task, given the wide variety of cancer types associated with the syndrome. Early diagnosis in patients during cancer treatment and their possible affected family members increases survival in both cases.

Genetic counseling and cancer screening under the Toronto Protocol (Table 2) proved to be cost-effective in Brazil [1]. Due to the greater risk of developing secondary neoplasms when exposed to ionizing radiation, rapid annual whole-body MRI allows the diagnosis of malignant neoplasms with asymptomatic carriers of the TP53 germline mutation, reducing the need for repeated tomography scans [1].

### 6.1. Radiotherapy, LFS, and Angiosarcoma

Angiosarcoma is a subtype of sarcoma arising from vascular or lymphatic endothelial cells, with aggressive behavior and higher incidence in elderly people between 60 and 90 years old. Its association with radiotherapy is well documented, particularly at doses exceeding 40 Gy, accounting 0.07% of cases in older series or conditions like chronic lymphedema. The incidence peaks between 8 and 10 years after initial treatment. The prognosis of patients with angiosarcoma associated with radiotherapy is poor, with an estimated 5-year survival rate of 43% [11]. However, interpreting data on such very rare diseases is extremely complicated. In the past, the absence of more qualified neoadjuvant or adjuvant treatments, delayed diagnosis, and comorbidities in a group with a more advanced median age at diagnosis certainly have compromised the prognosis. Currently, the higher rates of localized breast cancer at diagnosis and the consequent increase in patient survival may lead to more reported cases of late-onset angiosarcomas.

There is no specific histopathological definition to determine whether a sarcoma arising in an irradiated field is a primary or secondary malignancy, although the morphology of adjacent tissues may be suggestive if it shows radiation-related changes. MYC amplification at the 8q24.21 locus is a finding that can help define secondary angiosarcomas, as it is not seen in primary tumors [12].

Angiosarcomas associated with LFS outside irradiated areas are very rare findings, restricted to a few reports, but their presence in LFS patients associated with previous radiotherapy strongly suggests the syndrome. Women who received radiation therapy (RT) as part of their initial treatment for breast cancer faced a 16-fold increased risk of angiosarcoma and a twofold increased risk of other sarcomas [11]. These differences may indicate distinct mechanisms of radiation-induced carcinogenesis between angiosarcoma and other sarcomas. RT may directly increase the risk of angiosarcoma through radiogenetic mutations in irradiated tissues, and it is also possible that it indirectly contributes to the development of lymphedema. p53 plays a critical role in managing cellular responses to genotoxic damage, regulating the activation of downstream genes associated with apoptosis, cell cycle arrest, and DNA repair, and its deficiency predisposes a higher risk of new malignancies.

In the present case, some elements known today could help to indicate LFS: the young age at diagnosis of bilateral breast cancer, in addition to the strongly expressed HER2+ profile. Families with LFS often present soft tissue and bone sarcomas, particularly those with aneuploid karyotypes.

### 6.2. Clinical and Molecular Diagnosis of LFS

Screening of LFS in young individuals with breast cancer and no family history of cancer with multigene tests has been more encouraged than in the past. There is a trend toward testing all breast cancer patients under 65 years of age, regardless of family history [13]. This will undoubtedly detect an increasing number of pathogenic germline variants, especially in cases of poorly informative or absent family history, new mutations, mosaicism, or even variants of moderate penetrance that may be underestimated [13].

The pathogenic variant found in the case also deserves separate consideration. Missense variants form the focus of most cancer-relevant studies, and their properties have been investigated in numerous animal models. However, an important category of variants in TP53, which includes deletions, insertions, and duplications that alter the reading frame (frameshift) of TP53, represents another aspect of genomic alterations that remains poorly studied.

Like missense variants, most frameshift variants occur within the DBD. Several frameshift variants located in the DBD may not be detectable at the protein level due to the degradation of messenger RNA, caused by mRNA decay mediated by nonsense mutations (nonsense-mediated mRNA decay (NMD)). Others, located in downstream domains, can accumulate significantly within the cell. Two mutant constructs at Codon 345 (p.Asn345fs⁣^∗^26 and p.Asn345⁣^∗^) with preserved DBD were described showing altered oligomerization properties. Despite the high level of expression, they are mainly inactive and unable to initiate stimulus-induced transcriptional response, characteristic of wild-type TP53. However, one of these variants, p.Ile332fs⁣^∗^14, which resembles the naturally expressed *β* and *γ* isoforms, retains some residual antiproliferative activity and can induce cellular senescence in HCT116 cells. Cells expressing this mutant also exhibit decreased motility in migration assays. Therefore, this variant presents a combination of loss- and gain-of-function characteristics, distinguishing it from both wild-type TP53 and loss of TP53. This suggests two possible results of TP53 inactivation caused by frameshift variants: loss of antiproliferative activity (related or not to transcription, total or partial) and gain of function [14].

**Figure 3:**
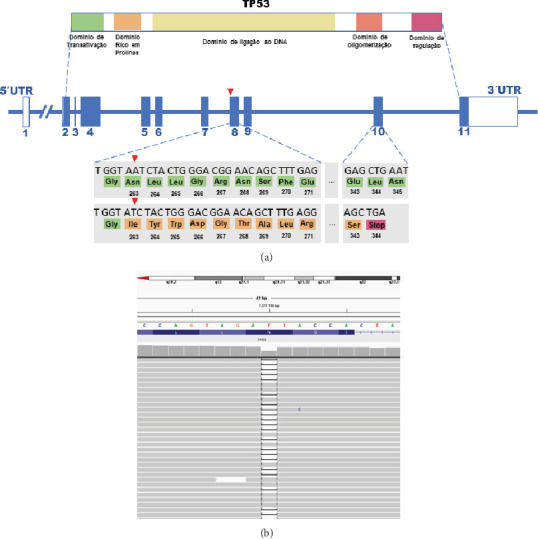
(a) The 11 exons of the TP53 gene and the encoded p53 protein domains. Exons 5 to 9 encode the most important domain of the protein, the DNA-binding domain, and are those most affected by pathogenic variants. (b) The patient's variant is in Exon 8. The deletion at Position 788 of an adenine can be observed. This change leads to the formation of a stop codon in Exon 10, 82 bases after the deletion. Therefore, the DNA binding site is partially dysfunctional, and the premature termination of the protein should produce a faster decay of TP53 mRNA.

The genetic test performed on the presented patient, using a multigene panel, revealed the presence of the pathogenic variant c.788del in heterozygosity in the TP53 gene. This change is of the frameshift type and corresponds to the deletion of an adenine at Position 788 of the coding region, resulting in the loss of the reading phase and the creation of a premature stop codon p.Asn263Ilefs⁣^∗^82. This deletion occurs in Exon 8, located in the DBD, but the premature stop codon occurs 82 amino acids downstream, in Exon 10, already in the OD (Figure 3). Therefore, loss of function is expected to occur due to premature truncation or mRNA decay mediated by nonsense mutations (NMD).

The c.788del variant is described in the ClinVar database as a pathogenic variant (SCV000936547.6) but is not included in other databases such as LOVD (Leiden Open (source) Variation Database) or the IARC TP53 germline variant bank. It is also not described in cases in the scientific literature. Other frameshift variants also with premature stop codons culminating in Exon 10 have previously been detected in families meeting classical criteria for LFS or Chompret syndrome [15] or are deposited as pathogenic variants in the ClinVar database (SCV001421075.3, SCV004460539.1, and SCV004286036.1).

There are no functional studies carried out for this variant or similar variants that cause the substitution of amino acids in the DBD, culminating in the creation of a premature stop codon in the OD. It is known that the exact location of the frameshift variant likely determines the physical and biological characteristics of the resulting protein, potentially impacting the protein's stability, its oligomeric status, intracellular localization, and DNA-binding properties. Therefore, it is not possible to estimate the effect of the detected change on the patient.

## 7. Conclusion

The case studied allowed the review of several issues involved in rare contexts like the one presented but which apply to a much larger number of patients. Multigene panels are increasingly necessary regardless of the patient's family history being suggestive of clustering of cancer cases or not.

Therapeutic choice and family cascade testing are fundamental elements that have not been widely accessible, regardless of socioeconomic context. In fact, missense variants are much more studied and common than indel or frameshift variants, and it is perfectly possible that different molecular changes produce different phenotypes. Thorough investigation and early diagnosis are crucial. Close monitoring by multidisciplinary teams at specialized centers can enhance the prognosis for affected patients.

## Figures and Tables

**Table 1:** Description of established clinical classification criteria for LFS.

**Criteria**	**Description**
Classic	Proband was diagnosed with sarcoma before the age of 45, and a first-degree relative with cancer before age 45, and another first or second-degree relative with any type of cancer diagnosed before the age of 45 or with sarcoma at any age

Chompret	Proband with sarcoma, brain tumor, breast cancer, or adrenal carcinoma before the age of 36, and at least one first- or second-degree relative with cancer (except breast cancer if the proband has breast cancer) under the age of 46, or a relative with multiple primary tumors at any age, or a proband with multiple primary tumors, two of which are sarcoma, brain tumor, breast cancer, and/or adrenal carcinoma, with the initial cancer occurring before the age of 36, regardless of family history, or a proband with adrenal carcinoma at any age of onset, regardless of family history

Birch	Among the families that do not conform to classic LFS: Proband with any childhood cancer or sarcoma, brain tumor, or adrenal carcinoma diagnosed under the age of 45, and a first- or second-degree relative with a typical LFS-related cancer (sarcoma, breast cancer, brain tumor, leukemia, or adrenal carcinoma) diagnosed at any age, and a first- or second-degree relative of the same genetic lineage with any type of cancer diagnosed under age 60

Eeles	Among the families that do not conform to classic LFS: Two different tumors that are part of the extended LFS in first- or second-degree relatives at any age (sarcoma, breast cancer, brain tumor, leukemia, adrenal tumor, melanoma, prostate, and pancreatic cancer)

**Table 2:** Toronto Protocol–recommended cancer surveillance for adults with TP53 pathogenic variant.

General evaluation	• Full physical checkup every 6 months

Breast cancer	• Breast exam twice a year starting at 20 years old • Breast MRI every year from 20 to 75 years • Risk-reducing mastectomy should be considered

Brain tumor	• Brain MRI every year, the first of which is contrast MRI • Subsequently, contrast is not necessary if the previous MRI is normal, and no new abnormalities are confirmed

Bone and soft tissue tumor	• Whole-body MRI every year • Abdominal and pelvic ultrasound every 12 months

Gastrointestinal	• Upper and lower GI endoscopy every 2–5 years

Malignant melanoma	• Dermatological examination every year

## Data Availability

The authors have nothing to report.
